# mTOR signaling is required for phagocyte free radical production, GLUT1 expression, and control of *Staphylococcus aureus* infection

**DOI:** 10.1128/mbio.00862-24

**Published:** 2024-05-20

**Authors:** Christopher J. Genito, Benjamin P. Darwitz, Callista P. Reber, Nathaniel J. Moorman, Christina L. Graves, Andrew J. Monteith, Lance R. Thurlow

**Affiliations:** 1 Division of Oral and Craniofacial Health Sciences, Adams School of Dentistry, University of North Carolina at Chapel Hill, Chapel Hill, North Carolina, USA; 2 Department of Microbiology and Immunology, School of Medicine, University of North Carolina at Chapel Hill, Chapel Hill, North Carolina, USA; 3 Department of Microbiology, University of Tennessee, Knoxville, Tennessee, USA; Universite de Geneve, Geneva, Switzerland; University of Illinois Urbana-Champaign, Urbana, Illinois, USA

**Keywords:** *Staphylococcus aureus*, mTOR, immune dysfunction

## Abstract

Mammalian target of rapamycin (mTOR) is a key regulator of metabolism in the mammalian cell. Here, we show the essential role for mTOR signaling in the immune response to bacterial infection. Inhibition of mTOR during infection with *Staphylococcus aureus* revealed that mTOR signaling is required for bactericidal free radical production by phagocytes. Mechanistically, mTOR supported glucose transporter GLUT1 expression, potentially through hypoxia-inducible factor 1α, upon phagocyte activation. Cytokine and chemokine signaling, inducible nitric oxide synthase, and p65 nuclear translocation were present at similar levels during mTOR suppression, suggesting an NF-κB-independent role for mTOR signaling in the immune response during bacterial infection. We propose that mTOR signaling primarily mediates the metabolic requirements necessary for phagocyte bactericidal free radical production. This study has important implications for the metabolic requirements of innate immune cells during bacterial infection as well as the clinical use of mTOR inhibitors.

**IMPORTANCE:**

Sirolimus, everolimus, temsirolimus, and similar are a class of pharmaceutics commonly used in the clinical treatment of cancer and the anti-rejection of transplanted organs. Each of these agents suppresses the activity of the mammalian target of rapamycin (mTOR), a master regulator of metabolism in human cells. Activation of mTOR is also involved in the immune response to bacterial infection, and treatments that inhibit mTOR are associated with increased susceptibility to bacterial infections in the skin and soft tissue. Infections caused by *Staphylococcus aureus* are among the most common and severe. Our study shows that this susceptibility to *S. aureus* infection during mTOR suppression is due to an impaired function of phagocytic immune cells responsible for controlling bacterial infections. Specifically, we observed that mTOR activity is required for phagocytes to produce antimicrobial free radicals. These results have important implications for immune responses during clinical treatments and in disease states where mTOR is suppressed.

## INTRODUCTION

Mammalian target of rapamycin (mTOR) has numerous important roles in regulating cell translation and metabolism. Upon activation, mTOR forms a complex with Raptor and several other proteins to form mTOR complex 1 (mTORC1), which then facilitates mRNA translation through ribosomal S6 kinase activation and inhibition of eukaryotic initiation factor 4E binding proteins (1). Through these functions, mTOR activation also facilitates protein, lipid, and nucleotide synthesis, which are key anabolic processes that support cell growth and proliferation.

Inhibitors of mTOR have widespread use in the treatment of human diseases. The mTOR inhibitor rapamycin (also known as Rapamune or Sirolimus; herein referred to as “Rapa”) has been FDA approved since 1999, originally to prevent organ rejection in renal transplant patients (2). Since then, Rapa or Rapa analogs (e.g., everolimus, tacrolimus, and temsirolimus) have been commonly used in the clinic for anti-rejection and cancer treatment. Numerous recent clinical trials have explored Rapa and its analogs in other clinical uses (3–8), including evaluation for the treatment of COVID-19 (9, 10).

Despite their diverse use as treatment in the clinic, Rapa and its analogs are classified as immunosuppressants. Indeed, their target mTOR is appreciated to have a role in immune activation. Toll-like receptor (TLR) and host cytokine signaling in response to infection activate mTOR through the PI3K/Akt pathway, with downstream effects on immune effector function (11). As such, it is well-known in the clinic that patients taking mTOR inhibitors have increased susceptibility to bacterial infection, often in the skin and soft tissues (12–16).

Phagocytes such as macrophages and neutrophils are key mediators of immune responses at barrier surfaces. As such, the immune functions of phagocytes are critical in the host response to bacterial skin and soft tissue infection (SSTI). A major mechanism of phagocytes to control bacterial infection is through the production of antimicrobial radical species, including radical oxygen species (e.g*.,* superoxide) and radical nitrogen species (e.g*.,* nitric oxide) (17). Individuals with genetic malfunction of phagocyte free radical production suffer from increased frequency and severity of SSTI (18). In bacterial infections, phagocyte activation through TLR and host cytokine signaling induces the production of free radicals through transcriptional mediators like NF-κB, STAT1, and IRF-1 (19, 20). A large influx of glucose is required to generate nicotinamide adenine dinucleotide phosphate (NADPH) to fuel nitric oxide and oxygen radical production through inducible nitric oxide synthase (iNOS) and phagocyte NADPH oxidase (NOX2) enzymatic activity, respectively (19–21). Phagocytes increase glucose uptake upon activation (22) and can maximize NADPH production from upper glycolysis by diversion through the pentose-phosphate pathway (PPP) (23). As metabolism through glycolysis and PPP are stimulated through mTOR signaling (24), it follows that susceptibility to bacterial infection mediated by mTOR inhibitors may be due to defects in radical oxygen and nitric oxide production by phagocytes.

*Staphylococcus aureus* is the most common cause of SSTI (25), and production of free radicals from phagocytes is required for clearance of *S. aureus* infection (26). We examine here the role of mTOR in the immune response using a murine model of *S. aureus* SSTI, where suppression of mTOR with Rapa treatment led to worse *S. aureus* infection. Treatment with Rapa did not alter local phagocyte presence or cytokine and chemokine production after week-long infection but inhibited free radical production in an iNOS- and NF-κB-independent manner. Phagocyte glucose transporter 1 (GLUT1) expression was found to be dependent on mTOR signaling, suggesting that mTOR is required for the glucose uptake required for free radical production. These experiments support a metabolic role for mTOR in the production of phagocyte free radicals and clearance of bacterial infection.

## RESULTS

### *S. aureus* burden and dissemination during infection

**Fig 1:**
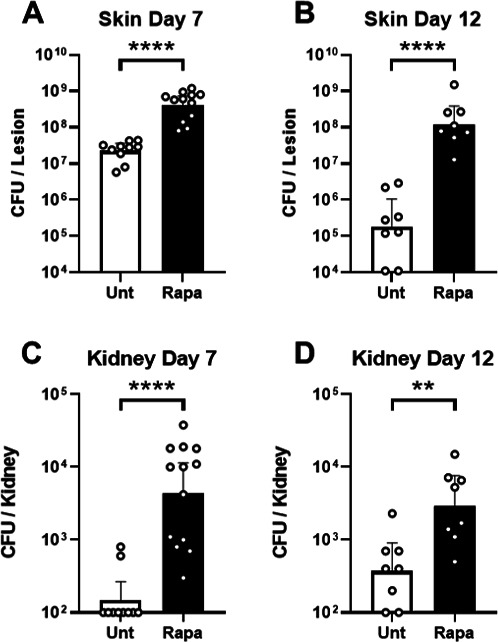
Suppression of mTOR during *S. aureus* SSTI leads to increased bacterial burden, greater dissemination, and impaired resolution of infection. *S. aureus* was isolated from the skin lesion (**A**) 7 days or (**B**) 12 days after infection, or isolated from the kidney (**C**) 7 days or (**D**) 12 days after subcutaneous infection in untreated (Unt) or rapamycin-treated mice. Bars represent geometric mean and 95% CI. **P < 0.01, ***P < 0.001, and ****P < 0.0001. Representative of three individual experiments.

To examine the role of mTOR signaling in controlling bacterial infection, mice were treated with the mTOR inhibitor rapamycin (Rapa) and inoculated subcutaneously with *Staphylococcus aureus* in a model of SSTI. *S. aureus* achieved a significantly higher burden in Rapa-treated mice than untreated mice (*P* < 0.0001), with ~20-fold more colony-forming units (CFU) recovered from the lesion on day 7 after infection in Rapa-treated mice (Fig. 1A). Suppression of mTOR with Rapa also severely inhibited the resolution of infection, as seen on day 12 where recovered CFU counts remained significantly higher in Rapa-treated mice (*P* < 0.0001; Fig. 1B). By day 12, CFU counts from the skin lesions of Rapa-treated mice had only fallen approximately threefold from day 7, remaining >10-fold higher than inoculum. The resolution was apparent in untreated mice on day 12, as CFU counts had fallen >100-fold from day 7. Observation of the kidneys from infected animals on day 7 revealed a significantly higher burden in Rapa-treated mice (*P* < 0.0001), with *S. aureus* detected in all animals (Fig. 1C). In contrast, *S. aureus* was detected in the kidneys of only two untreated mice. The amount of *S. aureus* recovered from the kidneys of Rapa-treated mice was similar between day 7 and day 12 (Fig. 1D). These experiments demonstrate a clear role for mTOR signaling in controlling bacterial burden, dissemination, and resolution of infection during *S. aureus* SSTI.

### Local phagocyte presence during infection

**Fig 2:**
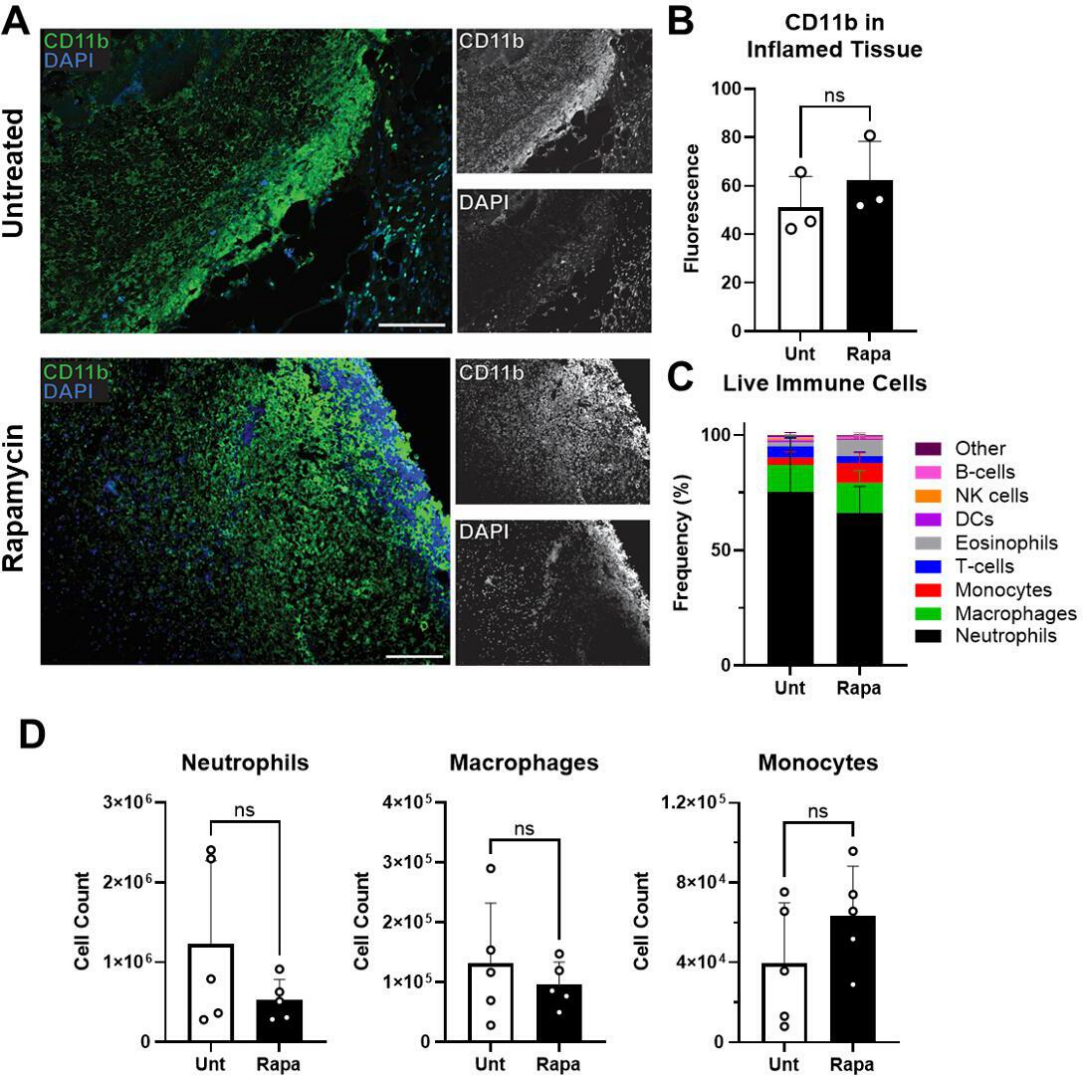
High degree of local phagocyte presence is maintained in *S. aureus* skin and soft tissue infection when mTOR is suppressed. Rapamycin-treated or untreated (Unt) mice were subcutaneously infected with *S. aureus* for 7 days and the resulting skin lesions were analyzed. (**A**) Fluorescent immunohistochemistry for CD11b expression (green; DAPI nuclear staining in blue) in tissue associated with skin lesion. Scale bar represents 50 µm at 20× magnification. (**B**) Average fluorescence intensity of CD11b quantified in IHC images. Each data point represents the analysis of an individual lesion. (**C**) Population frequencies of live immune cells within the lesion and surrounding inflamed tissue, quantified by flow cytometry. (**D**) Quantification by flow cytometry of neutrophils, macrophages, and monocytes in tissue associated with skin lesion. Bars represent mean and SD. Means not significantly different by *t*-test are denoted as “ns.” Representative of at least two independent experiments.

The increased bacterial burden during *S. aureus* infection in Rapa-treated mice strongly suggested that the immune response was compromised by mTOR inhibition. However, immunohistochemistry (IHC) revealed a similar abundance of CD11b^+^ cells present within the lesion between Rapa-treated and untreated mice during peak infection (Fig. 2A and B), and flow cytometric analysis also revealed a similar number of immune cells (Fig. S1 and S2A). Phagocytes (neutrophils, macrophages, monocytes, dendritic cells, and eosinophils) accounted for >90% of the live immune cells present in the lesion for both Rapa-treated and untreated mice (96% + 6% and 93% + 12%, respectively; Fig. 2C). The abundance of the most common phagocyte types (neutrophils, macrophages, and monocytes) was not significantly different between the two groups, though there was a modest increase in eosinophil presence in Rapa-treated mice (Fig. 2D; Fig. S1B). We also attributed a similar frequency and number of events during flow cytometric analysis of lesions from Rapa-treated and untreated mice to dead neutrophils (Fig. S2C and D). Importantly, the number of dead neutrophils was ~10-fold higher than live immune cells isolated from the lesion of both Rapa-treated and untreated mice, confirming that the immune response to the *S. aureus* SSTI was predominately through neutrophil recruitment. We concluded from these analyses that phagocyte presence during peak *S. aureus* SSTI was not reliant on mTOR signaling.

### Cytokine and chemokine signaling during infection

**Fig 3:**
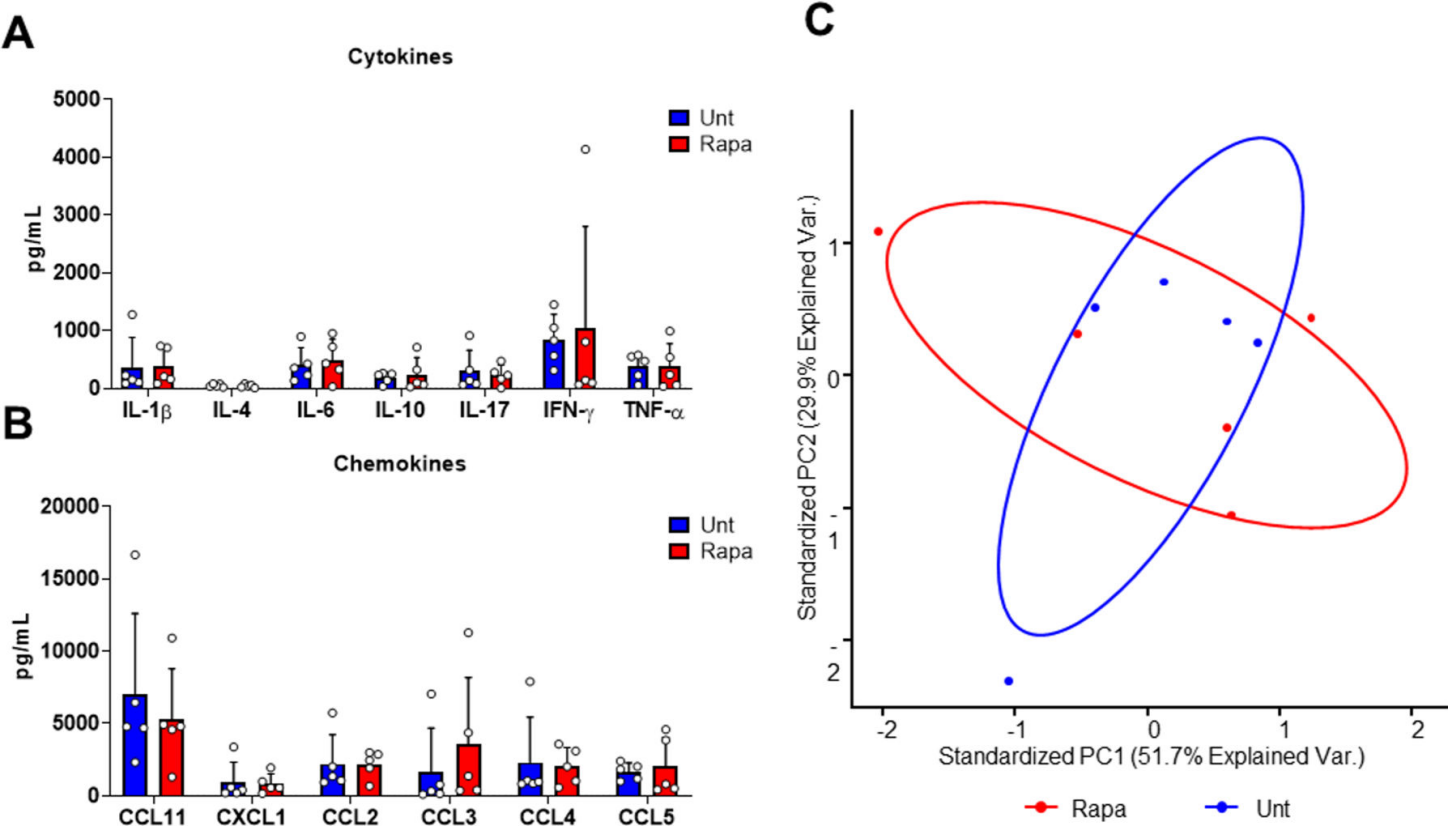
Cytokine and chemokine signaling is preserved in response to *S. aureus* skin and soft tissue infection during mTOR suppression. Selected (**A**) cytokines and (**B**) chemokines from multiplex protein analysis of resulting lesion 7 days after subcutaneous infection with *S. aureus* in untreated (Unt) or rapamycin-treated mice. Bars represent mean and SD. (**C**) Principal component analysis representing all 23 tested cytokines and chemokines.

As we determined that phagocyte presence in the *S. aureus-*infected lesion was largely unaffected by Rapa treatment, we turned our analysis to potential defects in immune function. We investigated the extent to which local immune signaling during SSTI may be impacted by mTOR inhibition at the site of infection. Multiplex analysis of 23 different cytokines and chemokines was performed on the lesion from *S. aureus*-infected mice during peak infection. However, we determined no significant differences between untreated and Rapa-treated mice for any of the tested cytokines and chemokines (Fig. 3A and B; Fig. S3) and a highly overlapping profile of protein levels (Fig. 3C). Thus, we concluded that mTOR signaling was not wholly essential for cytokine and chemokine signaling during infection.

### Phagocyte bactericidal activity and free radical production

**Fig 4:**
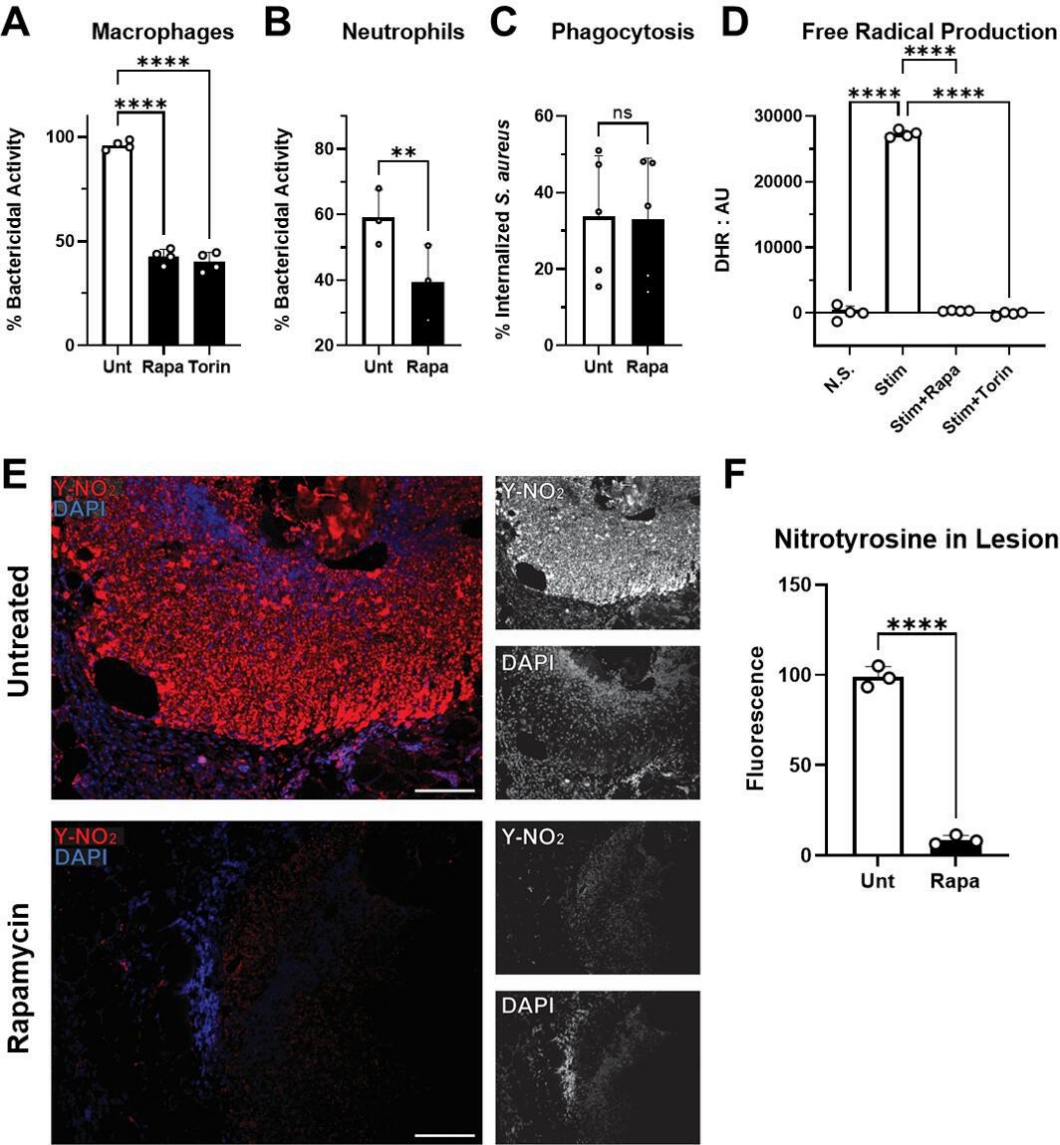
Phagocytes treated with mTOR inhibitors display decreased bactericidal activity and cannot produce superoxide and nitric oxide. *In vitro* bactericidal activity against *S. aureus* by (**A**) RAW264.7 macrophages and (**B**) human neutrophils treated with mTOR inhibitors rapamycin or Torin 2 (Torin), or untreated (Unt). (**C**) *In vitro* phagocytosis of *S. aureus* by human neutrophils in the presence of Rapa. (**D**) Detection of peroxynitrite, a by-product of nitric oxide and superoxide free radicals, by dihydrorhodamine (DHR) assay for RAW264.7 murine macrophages. N.S., not stimulated with LPS and IFN-γ. Stim, stimulated with LPS and IFN-γ. (**E**) Fluorescent immunohistochemical staining for nitrotyrosine (Y-NO_2_, red), a by-product of peroxynitrite, for lesions from *S. aureus* in rapamycin-treated or untreated mice after 7 days of subcutaneous infection. DAPI nuclear staining is shown in blue. Scale bar represents 50 µm at 20× magnification. (**F**) Average fluorescence intensity in nitrotyrosine staining images. Each data point represents the analysis of an individual lesion. Bars represent mean and SD. **P < 0.01, ****P < 0.0001, and ns, not significant (neutrophil data paired between individual donors, represented by each point in each group; comparisons with three or more groups use Dunnett’s correction for multiple comparisons to stimulated untreated). Data are representative of at least three individual experiments.

To further determine whether mTOR signaling was required for immune function during *S. aureus* infection, we performed an *in vitro* analysis of phagocyte bactericidal activity. Phagocytic macrophages cultured in the presence of two different mTOR inhibitors, Rapa or Torin, led to a significant decrease in bactericidal activity against *S. aureus* (Fig. 4A). Similar inhibition of bactericidal activity against *S. aureus* was observed when mTOR was inhibited in human neutrophils isolated from healthy donors (Fig. 4B). Importantly, suppression of mTOR was not associated with a decrease in uptake of *S. aureus*, showing no effect on phagocytosis directly (Fig. 4C). Neutrophil NETosis was also not affected by mTOR suppression (Fig. S4). However, treatment of phagocytes with mTOR inhibitors displayed no production of peroxynitrite, a by-product of nitric oxide and superoxide free radicals, upon activation with lipopolysaccharide (LPS) and interferon gamma (IFN-γ) (Fig. 4D). Taken together with the analysis of cytokine production during infection, we hypothesized that mTOR signaling is required for the control of *S. aureus* SSTI through the production of free radicals.

The nitrosylation reaction of peroxynitrite with tyrosine was readily observed by immunofluorescence in the *S. aureus*-infected lesions of mice during peak infection, corresponding to the release of nitric oxide and superoxide from phagocytes in response to bacterial infection (Fig. 4E and F). However, minimal nitrosylation was observed when mTOR was suppressed with Rapa treatment, corresponding to the inhibition of free radical production. Taken together, these *in vitro* and *in vivo* experiments showed that a sufficient level of mTOR signaling is required for phagocyte free radical production and bactericidal activity in response to *S. aureus* infection.

### Infection with nitric oxide-sensitive *Staphylococcus*

**Fig 5:**
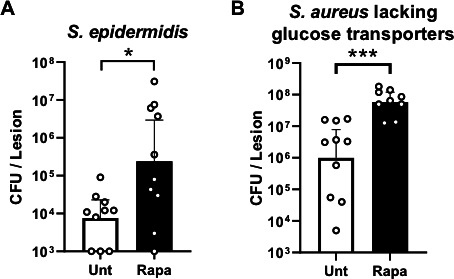
SSTI with free radical-sensitive *Staphylococcus* displays increased bacterial burden in mice treated with mTOR inhibitor. (**A**) Wild-type nitric oxide-sensitive *Staphylococcus* (*S. epidermidis*) or (**B**) mutant *S. aureus* lacking glucose transporters (upstream of lactate production to inhibit nitric oxide) was isolated from the lesion 7 days after subcutaneous infection in untreated (Unt) or rapamycin-treated mice. Bars represent geometric mean and 95% CI. *P < 0.05 and ***P < 0.001. Data are representative of two individual experiments.

To confirm the importance of mTOR-mediated free radical production in SSTI, we infected Rapa-treated mice with *Staphylococcus epidermidis,* a *Staphylococcus* species with sensitivity to phagocyte nitric oxide production and low infection potential (27, 28). Indeed, infection with *S. epidermidis* in the skin of mice was readily cleared (Fig. 5A). However, >30-fold more *S. epidermidis* CFUs were recovered from mice treated with Rapa. Thus, clearance of this phagocyte free radical-sensitive *Staphylococcus* species was significantly slowed when mTOR signaling was suppressed.

*S. aureus* expresses four designated glucose transporters, including two more high-affinity glucose transporters than other *Staphylococcus* species, including *S. epidermidis,* that confer resistance to nitric oxide produced by host phagocytes (29, 30). The increased glycolytic flux from these additional glucose transporters contributes to the lactate production involved in *S. aureus* resistance to nitric oxide (28–33). An *S. aureus* mutant lacking all four of its designated glucose transporters (29, 31) was observed to be attenuated during infection in untreated mice, showing no significant growth above inoculum (Fig. 5B). The low level of mutant *S. aureus* CFUs recovered from the lesions of untreated mice suggested that the infection was already being controlled and cleared. In contrast, the *S. aureus* mutant strain had significantly replicated above inoculum in Rapa-treated mice (*P* = 0.007), and ~60-fold higher CFUs were recovered from the lesion relative to lesions from untreated mice (Fig. 5B). The clear defect in the control of nitric oxide-sensitive *Staphylococcus* when mTOR is suppressed demonstrates that mTOR signaling plays a central role in free radical-dependent clearance of bacterial infection.

### Expression of iNOS and p65 nuclear translocation

**Fig 6:**
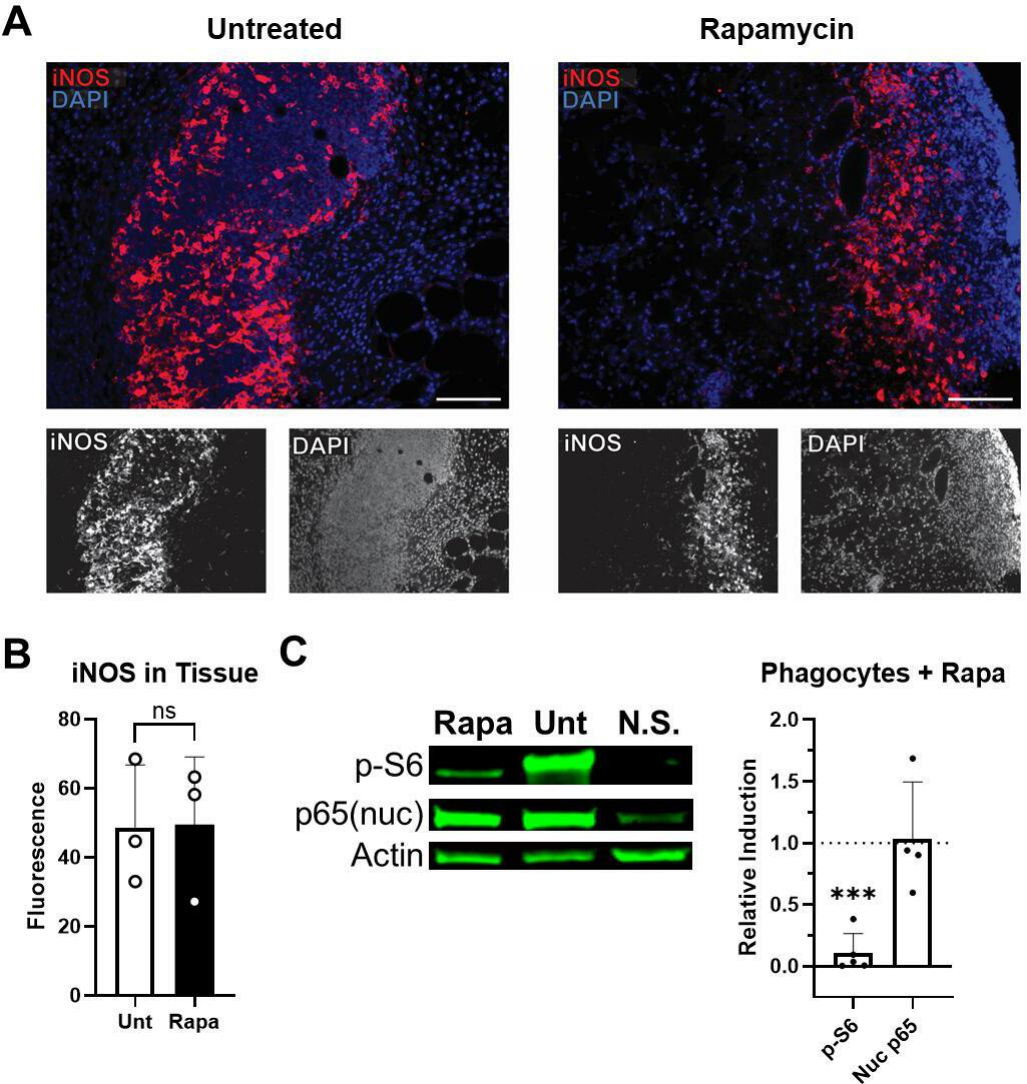
iNOS expression and p65 nuclearization in phagocytes are maintained during inflammatory conditions when mTOR is suppressed. (**A**) Tissue associated with lesions from rapamycin-treated or untreated mice (Unt) 7 days after subcutaneous *S. aureus* infection was analyzed by fluorescent immunohistochemistry for iNOS expression (red). DAPI nuclear staining is shown in blue. Scale bar represents 50 µm at 20× magnification. (**B**) Average fluorescence intensity of iNOS quantified in IHC images (ns, not significantly different means by *t*-test). Each data point represents the analysis of an individual lesion. (**C**) RAW264.7 murine macrophages were analyzed by Western blot for phosphorylated S6 (**P-S6**), pan actin (Actin), and nuclear (nuc) p65 protein levels 2 h after activation with LPS and IFN-γ. Relative protein induction upon activation is shown for rapamycin-treated macrophages relative to untreated macrophages. N.S., macrophages not stimulated with LPS and IFN-γ. Bars represent mean and SD; each point represents an individual experiment. ***P < 0.001, one-sample *t*-test compared to 1 (the value of 1 represents the level of protein induction of untreated macrophages upon stimulation).

A lack of nitrosylation in the lesion of Rapa-treated mice and increased susceptibility to nitric oxide-sensitive *Staphylococcus* infection suggested that nitric oxide was not being produced by phagocytes. However, we observed that iNOS was readily expressed in both untreated and Rapa-treated mice (Fig. 6A and B). As NF-kB signaling controls iNOS expression, Western blot analysis was performed to detect NF-κB subunit p65 in the nuclear extracts of phagocytes treated with Rapa (Fig. 6C; Fig. S5A through C). We observed no significant effect on p65 nuclear translocation by treatment with Rapa. These results indicated an iNOS- and NF-κB-independent role for mTOR signaling in nitric oxide release during infection.

### GLUT1 expression during infection

**Fig 7:**
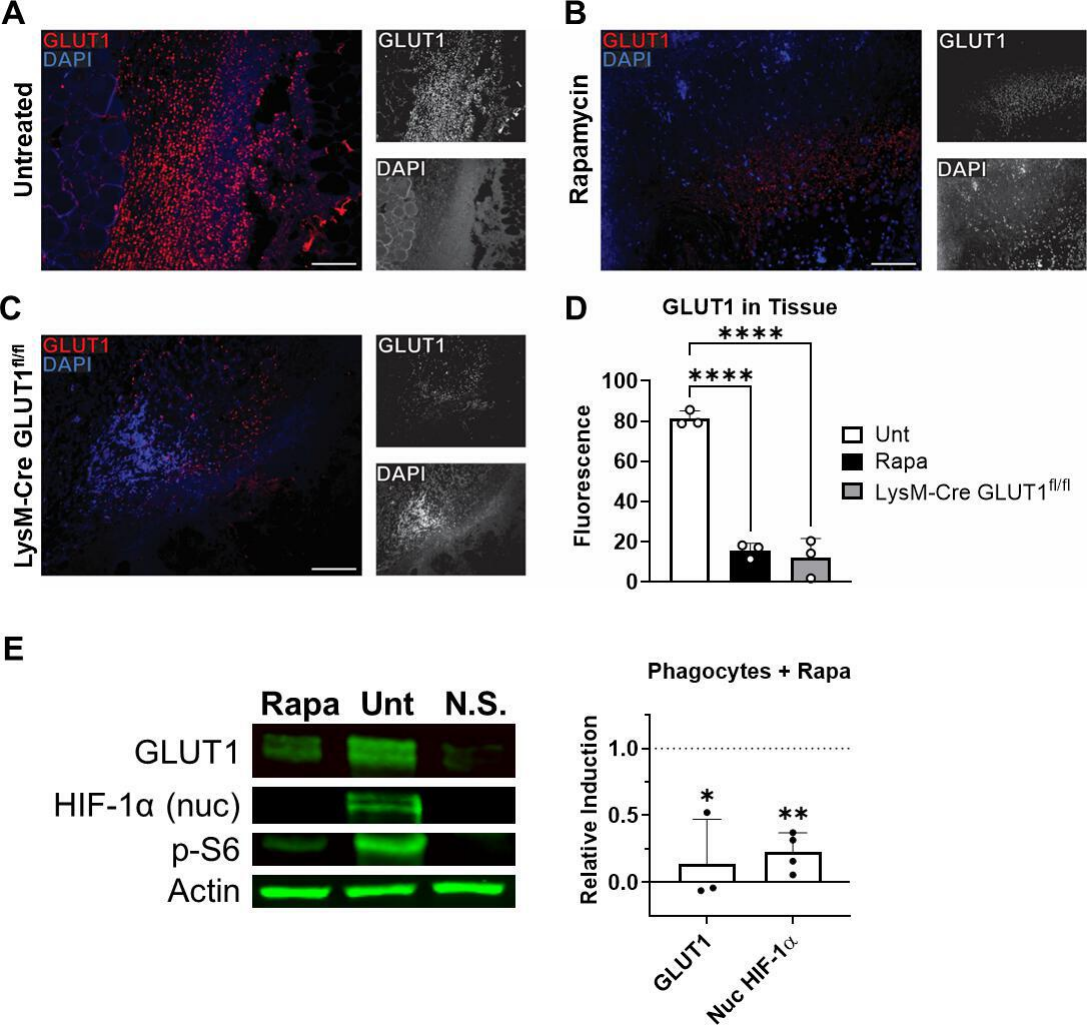
GLUT1 expression in mTOR-suppressed phagocytes is inhibited upon activation. (**A–C**) Tissue associated with lesions from mice 7 days after subcutaneous *S. aureus* infection was analyzed by fluorescent IHC for GLUT1 expression (red). Tissue was analyzed from infection in untreated (Unt) or rapamycin-treated wild-type mice and untreated LysM-Cre GLUT1^fl/fl^ mice. DAPI nuclear staining is shown in blue. Scale bar represents 50 µm at 20× magnification. (**D**) Average fluorescence intensity of GLUT1 quantified in IHC images. Each data point represents the analysis of an individual lesion. ****P < 0.0001, Dunnett’s multiple comparisons test to untreated. (**E**) RAW264.7 murine macrophages were analyzed by Western blot for GLUT1, nuclear (nuc) HIF-1α, phosphorylated S6 (**P-S6**), and pan actin (Actin) protein levels 2 h after activation with LPS and IFN-γ. Relative protein induction upon activation is shown for rapamycin-treated macrophages relative to untreated macrophages. N.S., macrophages not stimulated with LPS and IFN-γ. Bars represent mean and SD; each point represents an individual experiment. *P < 0.05 and **P < 0.01, one-sample *t*-test compared to 1 (the value of 1 represents the level of protein induction of untreated macrophages upon stimulation).

Generation of adequate amounts of NADPH is required for both nitric oxide and radical oxygen synthesis in phagocytes, which is produced by a large increase in glycolysis upon immune activation (34, 35). We observed that host glucose transporter GLUT1 expression was substantially diminished in the infected lesions of Rapa-treated mice during peak infection (Fig. 7A and B), similar to the extent to which GLUT1 is absent in the lesions of phagocyte-specific GLUT1 knock-out mice (LysM-Cre GLUT1^fl/fl^ mice; Fig. 7C and D). Western blot analysis revealed that the expression of GLUT1 upon activation was abrogated when Rapa-treated phagocytes were activated *in vitro* with LPS and IFN-γ (Fig. 7E; Fig. S5D). The transcription factor hypoxia-inducible factor 1α (HIF-1α) has been shown to control glycolytic capacity and GLUT1 expression during phagocyte activation, even under normoxic conditions (36). Nuclear HIF-1α accumulation was diminished when Rapa-treated phagocytes were activated *in vitro* (Fig. 7E; Fig. S5E). Data both from *in vivo* and *in vitro* supported that mTOR signaling induces GLUT1 expression during phagocyte activation, possibly through a HIF-1α-mediated manner.

**Fig 8:**
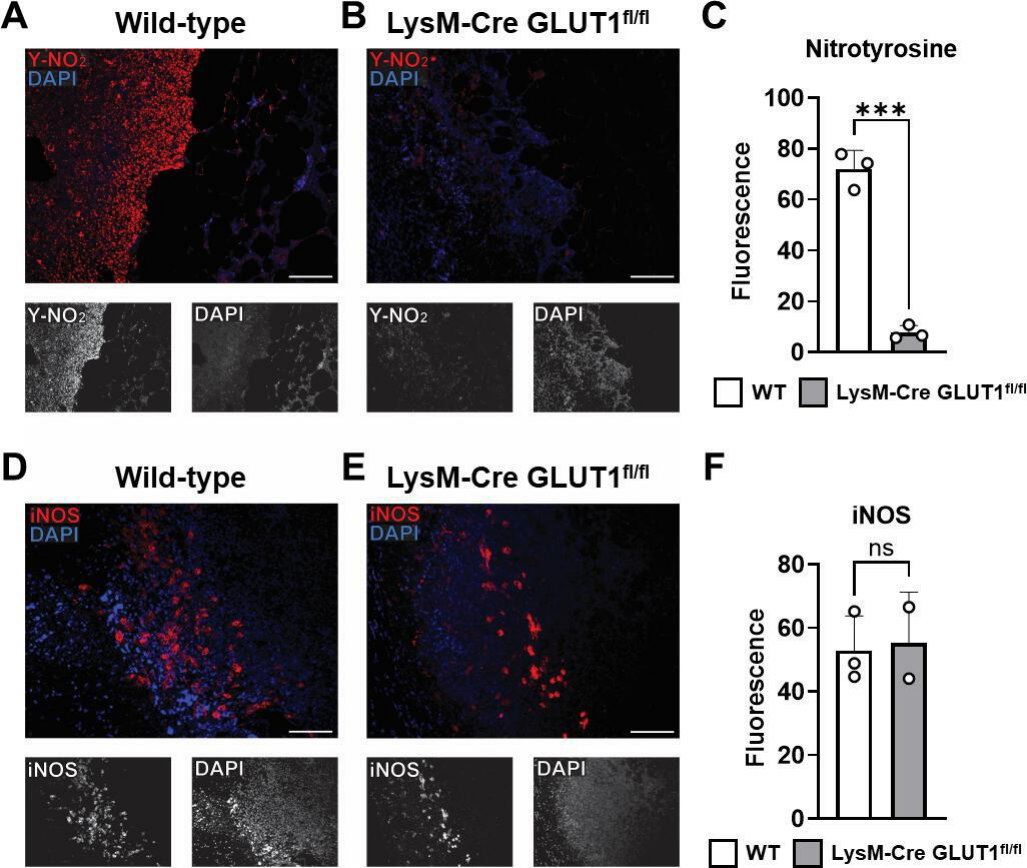
Free radical production is inhibited, but iNOS is preserved, in phagocyte-specific GLUT1-knockout mice. (**A and B**) Tissue associated with lesions from wild-type and LysM-Cre GLUT1^fl/fl^ mice 7 days after subcutaneous *S. aureus* infection was analyzed by fluorescent immunohistochemistry for nitrotyrosine (red). (**C**) Average fluorescence intensity of nitrotyrosine quantified in IHC images. (**D and E**) Analysis of tissue associated with lesions from wild-type and LysM-Cre GLUT1^fl/fl^ mice 7 days after subcutaneous *S. aureus* infection by IHC for iNOS (red). (**F**) Average fluorescence intensity of iNOS quantified in IHC images. DAPI nuclear staining is shown in blue. Scale bar represents 50 µm at 20× magnification. Each data point represents the analysis of an individual lesion. Bars represent mean and SD. ***P < 0.0001 and ns, not significant by *t*-test.

We have previously shown that conditional knock-out of GLUT1 from phagocytes in LysM-Cre GLUT1^fl/fl^ mice inhibits free radical production during *S. aureus* SSTI, leading to more severe infection (31). Similar to what was seen in Rapa-treated mice, iNOS expression was readily observed in the *S. aureus*-infected lesions of LysM-Cre GLUT1^fl/fl^ mice, though nitrosylation by peroxynitrite was impaired (Fig. 8). We therefore concluded that the mechanism of immune suppression that Rapa-treatment confers during *S. aureus* SSTI is due to the inhibition of mTOR-mediated GLUT1 expression upon phagocyte activation, which prevents the release of bactericidal free radicals.

## DISCUSSION

Clinically, mTOR inhibitors are classified as immunosuppressants. Indeed, inhibition of mTOR can dampen neutrophil effector function (37) and chemotaxis (38), block anti-viral IFN-α/β responses from plasmacytoid DCs (39), and promote the development of tolerogenic DCs (40, 41). However, mTOR signaling has a range of other diverse functions in immunity that are seemingly paradoxical to Rapa’s classification as an immunosuppressant. A role for mTOR signaling has been described in the promotion of Treg (42) and myeloid-derived suppressor cell function (43) and the regulation of T-cell memory (44). Rapa also has applications in the treatment of cancer to improve immune responses, reducing T-cell exhaustion (7, 45) and enhancing T-cell effector memory function and tumor infiltration (46). It is thought that the discrepancy between mTOR inhibitors causing immune suppression or stimulation depends on the administered dose, associated with higher or lower doses, respectively (47).

Rapa administered in our model was at a relatively high dose for mice and inhibited the immune system’s ability to control *S. aureus* infection. The mechanism of immune suppression presented here is that mTOR signaling is required for the phagocyte-mediated immune response to *S. aureus* infection. Specifically, mTOR activation was shown to enhance GLUT1 expression by phagocytes, potentially through activation of HIF-1α, enabling the production of bactericidal free radicals. GLUT1 expression then putatively mediates the activation-induced increase in glycolysis required for adequate NADPH production for free radical synthesis.

Multiple studies have supported a growing appreciation for the metabolic requirements of phagocyte activity during a typical immune response. Neutrophil production of free radicals *ex vivo* has been shown to be inhibited when treated with Rapa (37, 48), and GLUT1 overexpression in macrophages increases the production of reactive oxygen species (49). A recent article has connected eosinophil-mediated inflammation with *S. aureus* infection (50), and we observed a minor but significantly increased eosinophil population during *S. aureus* infection in Rapa-treated mice. It is unclear what role mTOR signaling may play in the eosinophil response during *S. aureus* SSTI. Our model denotes a role for mTOR signaling during a phagocyte-mediated immune response to both a virulent and attenuated mutant strain of *S. aureus*, as well as in response to *S. epidermidis*, which is considered a commensal species. The metabolic role for mTOR in phagocyte-mediated immunity explored here may have important applications in the immune response to other bacterial pathogens (e.g., *Pseudomonas aeruginosa* [51]), or perhaps fungal pathogens (e.g., *Candida albicans* [52]), that are sensitive to phagocytosis and free radical production.

Control of HIF-1α expression by mTOR has been previously established, as treatment with Rapa can inhibit HIF-1α in tumor cells (53). Further evidence also exists to support the role of HIF-1α in the production of free radicals from activated phagocytes, even in the absence of hypoxia. HIF-1α is upregulated in activated human macrophages (54), controls glycolytic capacity and GLUT1 expression in peritoneal macrophages (36), and facilitates nitric oxide production and bactericidal killing of *S. aureus* by phagocytes (55). We showed here a reduction in nuclear HIF-1α upon activation of phagocytes treated with Rapa under normoxic conditions, but it is still unclear whether HIF-1α signaling is required for phagocyte GLUT1 expression and free radical-mediated clearance of *S. aureus* infection in our model.

We show here that iNOS expression was still present in Rapa-treated mice during peak *S. aureus* infection. Host cytokines that stimulate iNOS expression through NF-κB signaling (e.g*.,* TNF-α and IL-1β [56]) and independently of NF-κB (i.e*.,* IFN-γ through STAT-1α and IRF-1 signaling [56]) were similar between Rapa-treated and untreated mice. We also observed that early activation-associated p65 nuclear translocation was not significantly different in mTOR-inhibited phagocytes. While both mTOR and NF-κB can be activated through the PI3K/Akt pathway in response to bacterial and host factors, our results suggest a separate, parallel function in the immune response to *S. aureus* SSTI where mTOR signaling supports metabolic functions of the phagocyte and NF-κB signaling controls cytokine production. Indeed, others have shown that GLUT1 knockout in phagocytes does not greatly impact cytokine production upon activation (49, 57). Crosstalk between mTOR and NF-κB signaling has been described, especially in tumor cells (58), and several studies have observed an increase in IL-12 production from Rapa-treated phagocytes upon activation (59–61), though we did not observe a significant difference in IL-12 production in the lesions of Rapa-treated animals. One explanation may be a potential override of mTOR/NF-κB crosstalk during infection with a *bona fide* pathogen in the context of a complex *in vivo* host immune response. It may also be important to note that phosphorylation of p65 at S536, which is often used to study NF-κB activation, may represent a non-canonical NF-κB pathway (62) and, therefore, may not be relevant to such a strong canonical activation of NF-κB during infection. Nevertheless, our results show that cytokine signaling and phagocyte presence during peak infection are not sufficient to compensate for the impaired production of free radicals from phagocytes in the control of *S. aureus* infection.

Our experiments with *S. aureus* infection in LysM-Cre GLUT1^fl/fl^ mice demonstrated that GLUT1 expression in phagocytes is required for the production of free radicals and control of infection. Previous investigations have observed inhibited free radical production from phagocytes and worse infection outcomes during *S. aureus* infection in a diabetic host (31, 63). We have previously established that the impaired free radical production and compromised immune response to *S. aureus* infection in a diabetic host were due to impaired phagocyte GLUT1 expression (31). Importantly, insulin signaling activates mTORC1 through the PI3K/Akt pathway (64). In the diabetic disease state where insulin signaling is impaired, control of GLUT1 expression by mTOR signaling shown here may connect impaired phagocyte GLUT1 expression to worse *S. aureus* infection outcomes in diabetes.

Control of *S. aureus* SSTI through the host immune response is dependent on mTOR signaling in phagocytes, primarily through allowing the production of bactericidal free radicals. The role of mTOR in the phagocyte immune response is metabolic, controlling the expression of host glucose transporter GLUT1, potentially through a HIF-1α-mediated mechanism, and putatively allowing the activation-induced increase in glycolysis to generate adequate NADPH for free radical production. Our *in vivo* experiments show that mTOR inhibition does not affect phagocyte presence, cytokine and chemokine production, or iNOS expression during peak infection. Combining these observations with *in vitro* experiments confirming similar p65 nuclear translocation, our data support a cooperative but separate role of mTOR signaling from NF-κB signaling within phagocytes in response to infection. Altogether, this study provides insight into Rapa-mediated increases in susceptibility to bacterial infections in the clinic and elucidates a metabolic role for mTOR in the immune response to bacterial pathogens. Further investigation of how mTOR signaling functions during infection in the context of metabolic disease states such as diabetes is of considerable clinical interest.

## MATERIALS AND METHODS

### Materials

Rapamycin was obtained from LC Laboratories. Torin 2 (Torin) was obtained from Cayman Chemical. LPS (from *Salmonella enterica* serotype enteritidis) was obtained from Sigma-Aldrich. Interferon-gamma was obtained from Peprotech. Antibodies used for immunohistochemistry, flow cytometry, and Western blot analyses are listed in Table S1.

### Mice and infection model

All animals used in this study were housed in an AAALAC-accredited facility and used according to an IACUC-approved protocol. Six- to eight-week-old female C57BL/6 mice were purchased from the Jackson Laboratory and housed at the University of North Carolina at Chapel Hill. LysM-Cre GLUT1^fl/fl^ mice were housed and bred at the University of North Carolina at Chapel Hill. Wild-type mice were treated with 8 mg Rapa/kg of body weight daily for 5 days, followed by 2 days of rest, and daily treatments were resumed starting on the day of infection. Skin and soft tissue infection was induced by subcutaneous 20 µL injection of 1 × 10^7^ CFU *S. aureus* (LAC strain) or 1 × 10^8^ CFU *S*. *epidermidis* (1457 strain).

### Fluorescent immunohistochemistry

Immunofluorescence staining was performed using de-paraffinized formalin-fixed tissue sections (10 µm) from the lesions of infected mice, as described previously (31, 65). Briefly, sections were blocked in 10% donkey serum for 1 h before overnight incubation with rabbit primary antibody. Samples were then incubated in biotinylated donkey anti-rabbit IgG secondary antibody and developed with DyLight 488-conjugated or Alexa Fluor 594-conjugated streptavidin (Jackson ImmunoResearch). After staining, sections were mounted using ProLong Gold Antifade Mountant with DAPI (Thermo Fisher Scientific) and imaged using an Olympus BX60 microscope and iVision software (BioVision Technologies). To quantify fluorescence in IHC images, average pixel intensity in areas of interest was measured relative to the background using Fiji (66).

### Spectral flow cytometry

Skin lesions from mice infected with *S. aureus* were removed and prepared for flow cytometric analysis by digesting with collagenase type I (Gibco, 1 mg/mL) and DNase I (Roche, 50 µg/mL) for 30 min at 37°C, mashing through a 70 µm nylon filter, treating with ACK lysis buffer, and then straining through a 40 µm nylon filter. Cells were counted and then stained with viability dye (Pacific Blue-conjugated succinimidyl ester, Life Technologies), blocked with TruStain FcX (BioLegend), incubated with primary antibody cocktail, and then fixed with BD Cytofix/Cytoperm. Spectral flow cytometry was performed using a Cytek Aurora and unmixed using the Cytek SpectroFlo software. Data analysis was performed using FlowJo (BD).

### Multiplex cytokine analysis

Abscesses from mice 7 days after the induction of *S. aureus* SSTI were homogenized in PBS containing 0.5% BSA and 1 mM EDTA, then centrifuged at 17,000 × *g* for 10 min. The supernatant was further diluted 1:20 and analyzed using the Bio-Plex Pro Mouse Cytokine 23-plex Assay (Bio-Rad). Interpolated cytokine concentrations were normalized to the total protein in each sample, determined by BCA assay.

### Culture of phagocytes

Human circulating neutrophils were isolated from the peripheral blood of healthy donors. Single-cell suspensions of blood were subjected to density centrifugation to obtain neutrophils at the interface between layers of Histopaque 1119 and Histopaque 1077. Prior to any experiments, isolated neutrophils were rested on ice for 45 min in D10 media (DMEM and 10% FBS) prior to being transferred to ultra-low attachment round-bottom 96-well plates in D10 medium to incubate (37°C, 5% CO_2_) for 30 min. Following incubation, neutrophils were treated with rapamycin (100 ng/mL) for 1 h. Data for isolated neutrophils were collected using an AttuneNxT flow cytometer with Attune software and analyzed using FlowJo. All samples were gated forward scatter height (FSC-H) by forward scatter area (FSC-A) to remove doublet populations and the singlet population was gated FSC-H by SSC-H to isolate the granulocyte population. RAW264.7 murine macrophage cell line (RAWs) was obtained from the UNC Tissue Culture Facility and grown in high-glucose DMEM supplemented with 1 mM sodium pyruvate, 4 mM L-glutamine, 10% FBS, and 1% penicillin/streptomycin. RAWs were plated at 1 million cells/well in tissue cultured-treated 6-well plates and incubated at 37°C, 5% CO_2_ until confluent (2–3 days).

### *In vitro* bactericidal assays

*In vitro* killing of *S. aureus* by RAWs was determined as previously described (31). Briefly, RAWs were incubated for 18 h in the presence of Rapa or Torin before activation with LPS and IFN-γ for 1 h. *S. aureus* (LAC strain) was then added to the culture at an MOI of 10:1 CFU to phagocyte. Opsonization was allowed for 30 min before removal of extracellular bacteria with gentamicin treatment. RAWs were lysed with 0.01% Triton X-100 after 12 h for the quantification of *S. aureus* CFU. Bactericidal activity was calculated using the resulting CFU relative to inoculum.

Human neutrophils were cultured with *S. aureus* (MOI = 0.5) in D10 media. Bactericidal activity was calculated after 4 h using the CFU count of the bacteria-immune cell cultures relative to the CFU count of cultures containing bacteria alone.

### Phagocytosis assay

Human neutrophils were cultured with a fluorescent strain of *S. aureus* (p*SarA_sfGFP*; MOI = 10), and 20 min before fixation, Ghost Dye Violet 510 (Tonbo Biosciences) was introduced to the cultures to identify live neutrophils. After neutrophils were cultured with *S. aureus* for 3 h, neutrophils were then fixed with 4% paraformaldehyde, blocked with Human TruStain FcX (BioLegend), and stained with anti-CD16 and anti-CD15 antibodies for analysis by flow cytometry. Granulocytes were gated for neutrophils (CD16- and CD15-positive). For total phagocytosis, the raw GFP MFI was quantified, and the percentage of neutrophils that phagocyted *S. aureus* was quantified relative to the total number of neutrophils.

### *In vitro* phagocyte free radical production

Respiratory burst analysis was performed using Dihydrorhodamine 123 (DHR, Cayman Chemical), as previously described (31). Briefly, RAWs were incubated for 24 h at 37°C, 5% CO_2_ in the presence of Rapa (100 ng/mL) or Torin (100 nM), then activated with LPS and IFN-γ for 1 h. DHR was added at the time of activation and detected by fluorescence.

### NETosis assay

Isolated human neutrophils were cultured with *S. aureus* (MOI = 10), and 20 min before fixation, Ghost Dye Violet 510 (Tonbo Biosciences) and Sytox Blue (Invitrogen) were introduced to the cultures to identify neutrophils with compromised membranes or extracellular DNA, respectively. After neutrophils were cultured with *S. aureus* for 3 h, neutrophils were fixed with 4% paraformaldehyde, blocked with Human TruStain FcX (BioLegend), and stained with anti-CD16 (BioLegend), anti-CD15 (BioLegend), anti-MPO-Biotin (Abcam), and anti-H3Cit (Abcam) antibodies. Secondary staining was performed with fluorescent anti-rabbit IgG and streptavidin. Granulocytes were gated for neutrophils (CD16- and CD15-positive), and neutrophils with permeabilized cell membranes that were positive for extracellular dsDNA, MPO, and H3Cit were defined as having undergone suicidal NETosis as previously described (67). The percentage of neutrophils undergoing suicidal NETosis was quantified relative to the total number of neutrophils.

### Analysis of phagocyte protein induction by Western blot

Activation of phagocytes for protein analysis was performed with LPS (25 ng/mL) and IFN-γ (20 ng/mL) for 2 h. RAWs were treated with Rapa at the time of activation and 18–24 h preceding. Cell lysates for S6 analyses were obtained with radioimmunoprecipitation assay buffer. NE-PER Nuclear and Cytoplasmic Extraction Reagents (Thermo Fisher Scientific) were used to obtain nuclear extracts for the analysis of nuclear NF-κB subunit p65 and nuclear HIF-1α, as well as cytosolic GLUT1. Halt protease and phosphatase inhibitor cocktail (Thermo Fisher Scientific) and 5 mM EDTA were used in all extractions. Cell lysates were heated for 5 min at 100°C (except samples for GLUT1 analysis, which were kept at RT) in the presence of protein sample loading buffer (LI-COR) and 100 mM DDT. A volume of 20 µL/well was loaded onto a 12% polyacrylamide gel, and electrophoresis was run for 90 min at 100 V. Gels were blotted onto nitrocellulose using an iBlot2 (Thermo Fisher Scientific) and blocked at RT with Intercept (TBS) blocking buffer (LI-COR) for 2 h before incubating in rabbit primary antibody overnight. Blots were developed with 1:15,000 IRDye 800CW goat anti-rabbit IgG secondary antibody (LI-COR) for 1 h and imaged on a LI-COR Odyssey DLx. Densitometry was performed using LI-COR Empiria Studio software. Band intensity was normalized to actin band intensity and expressed as relative to unstimulated control. Analysis of phosphorylated S6 was performed in all Western blot experiments to confirm Rapa-mediated mTOR suppression. Analysis of total S6 protein was performed to confirm no changes in total S6 production by Rapa treatment.

### Statistical analysis

Statistical significance in comparisons between groups was determined with GraphPad Prism 10 software. Comparisons between two groups were made with a *t*-test. Comparisons between three or more groups were made with ANOVA and correction for multiple comparisons. Data involving human neutrophils were averaged between two to three technical replicates, and comparisons were paired between samples from the same individual donor. CFU counts were log-transformed for analysis. Principal component analysis was performed using the R function prcomp() and plotted with ggbiplot().

## Data Availability

All data are available in the text or the supplemental material.

## SUPPLEMENTAL MATERIAL

Supplemental material mbio.00862-24-s0001.pdf Figures S1 to S5 and Table S1.

ASM does not own the copyrights to Supplemental Material that may be linked to, or accessed through, an article. The authors have granted ASM a non-exclusive, world-wide license to publish the Supplemental Material files. Please contact the corresponding author directly for reuse.
