# Supplementary material for: mTOR signaling is required for phagocyte free radical production, GLUT1 expression, and control of Staphylococcus aureus infection
Source: mBio. 2024 May 20;15(6):e00862-24. doi: 10.1128/mbio.00862-24 (PMC11324022; doi:10.1128/mbio.00862-24)
Supplement: Supplemental material — Figures S1 to S5 and Table S1. [file mbio.00862-24-s0001.pdf]

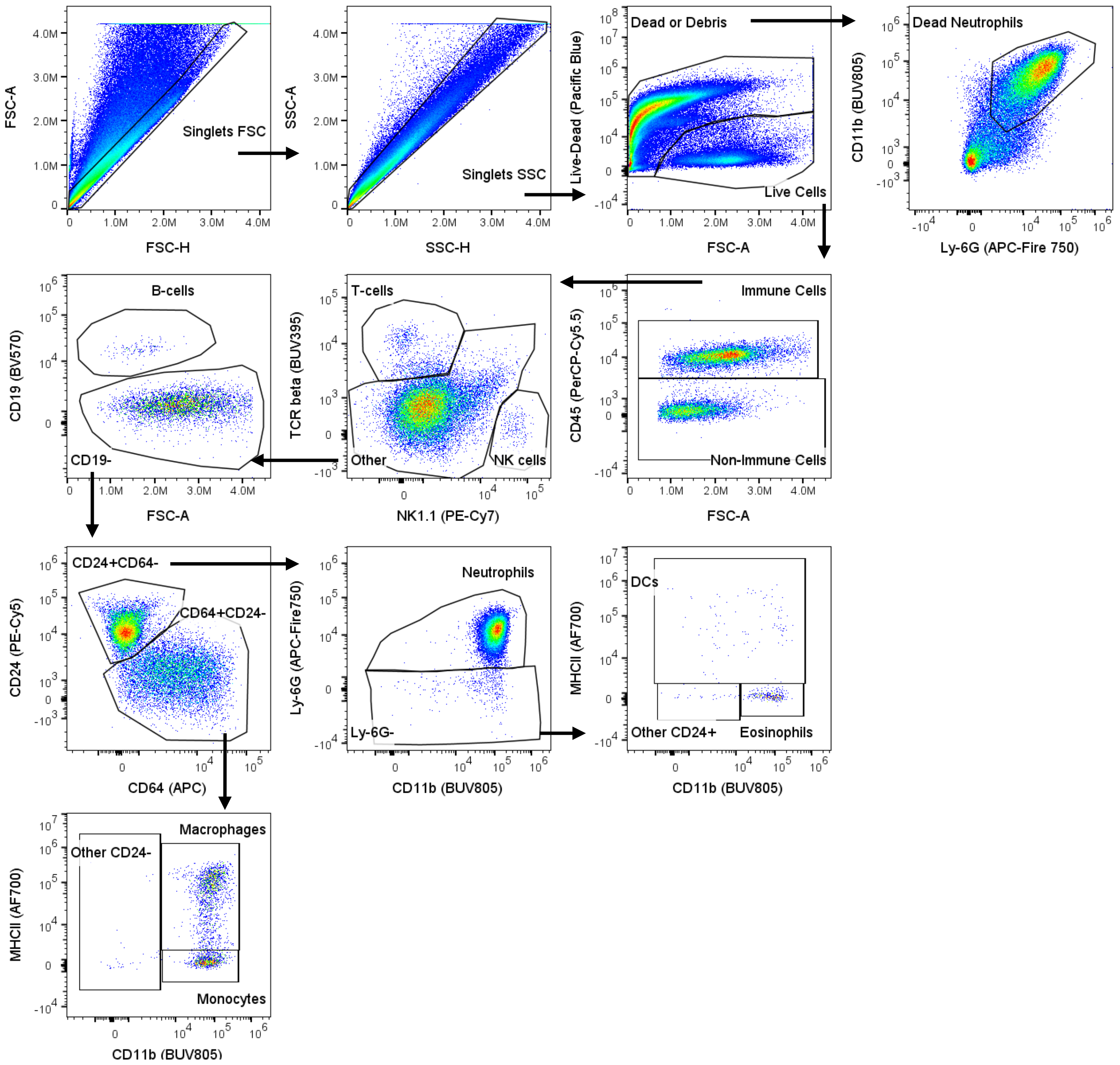

Figure S1. Gating Scheme for flow cytometry analysis of *S. aureus* skin and soft tissue infection.

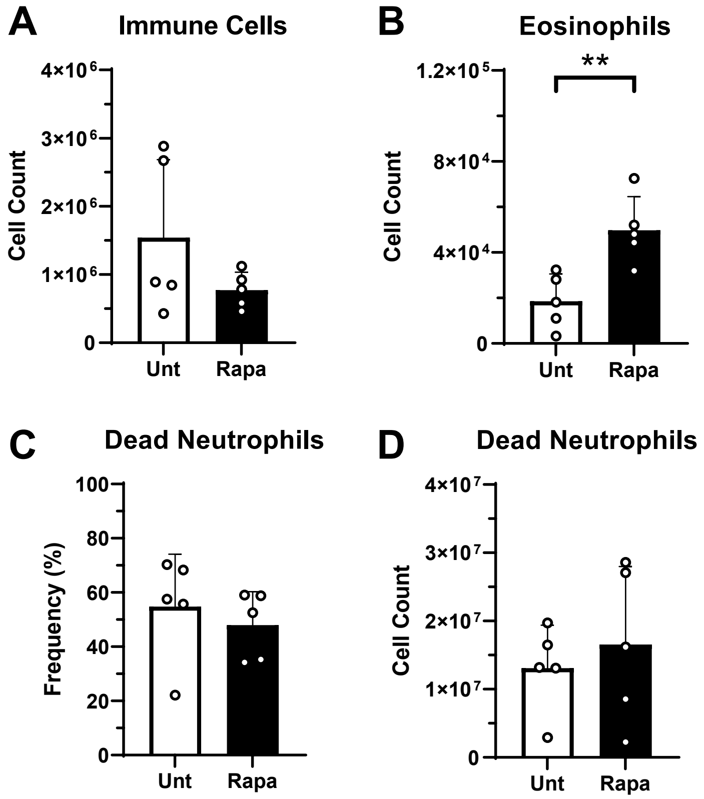

Figure S2. Additional immune cell populations analyzed by flow cytometry. Rapamycin-treated (Rapa) or untreated (Unt) mice were subcutaneously infected with *S. aureus* for 7 days. Resulting skin lesions were analyzed by flow cytometry. **(A)** Total immune cells quantified by CD45 expression. **(B)** Eosinophil count. **(C)** Frequency of cells expressing neutrophil markers among viability dye-positive singlet events. **(D)** Quantification of dead neutrophils. Bars represent mean and SD. $^{**}p < 0.01$.

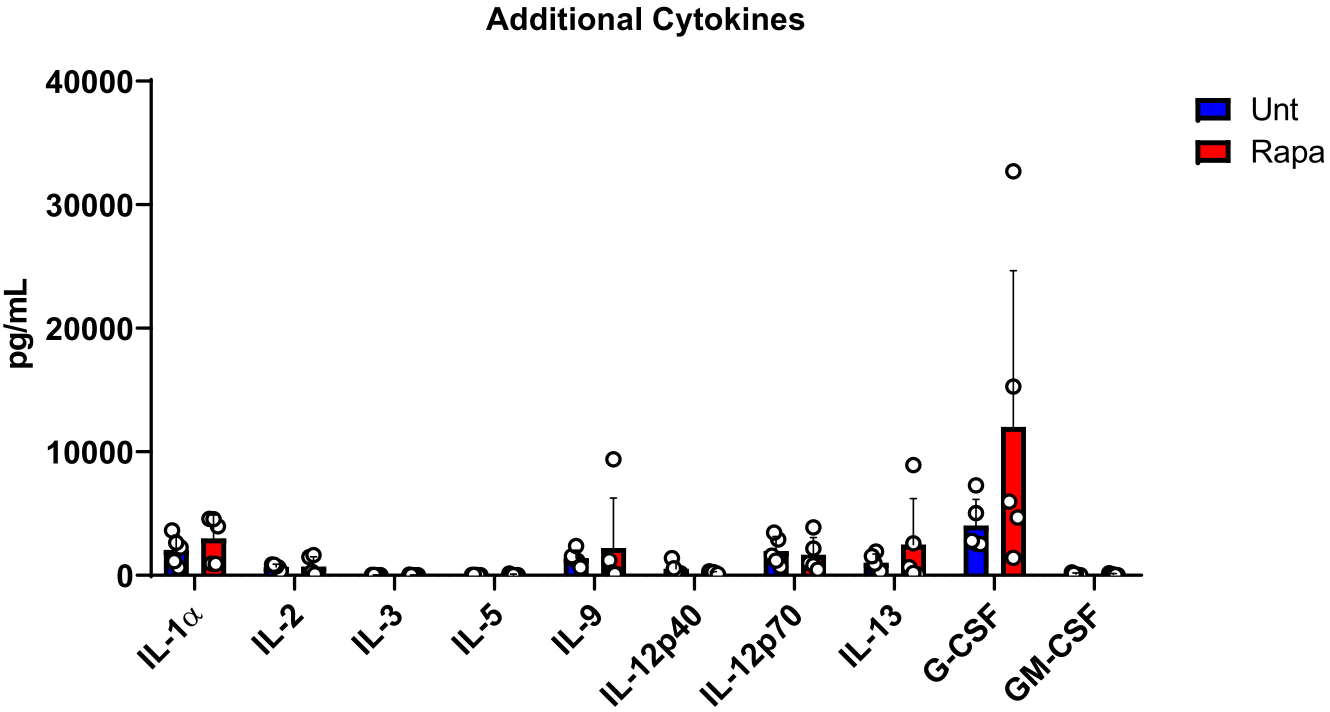

Figure S3. Additional cytokine levels preserved in response to *S. aureus* skin and soft tissue infection during mTOR suppression. Additional cytokine levels from 23-plex protein analysis of resulting lesion 7 days after subcutaneous infection with *S. aureus* in untreated (Unt) or rapamycin (Rapa)-treated mice. Bars represent mean and SD.

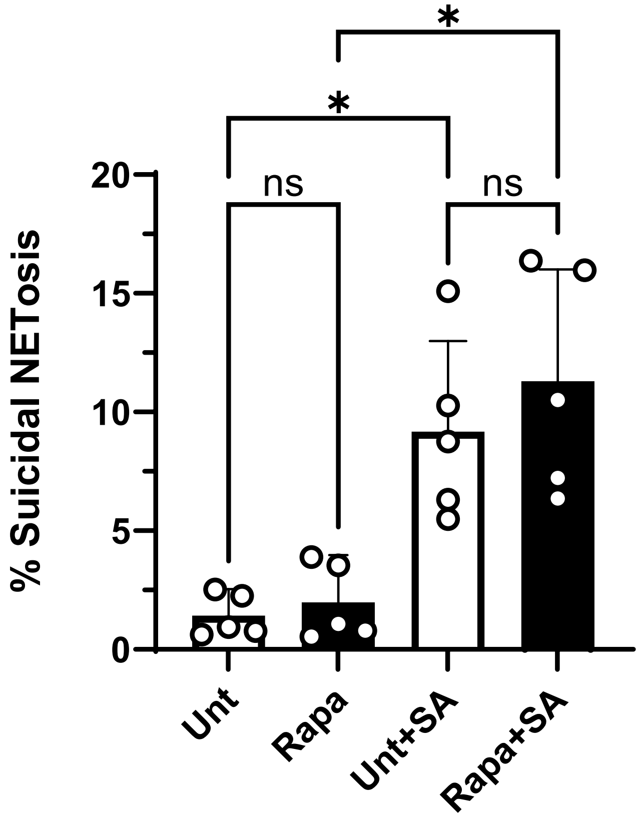

Figure S4. *In vitro* NETosis of neutrophils during mTOR suppression. *In vitro* NETosis assay of human neutrophils treated with mTOR inhibitor rapamycin (Rapa) or untreated (Unt). "+SA" denotes the presence of *Staphylococcus aureus.* Bars represent mean and SD. *$p<0.05$, ns = not significant (data paired between individual donors, represented by each point in each group; Šídák's correction for multiple comparisons).

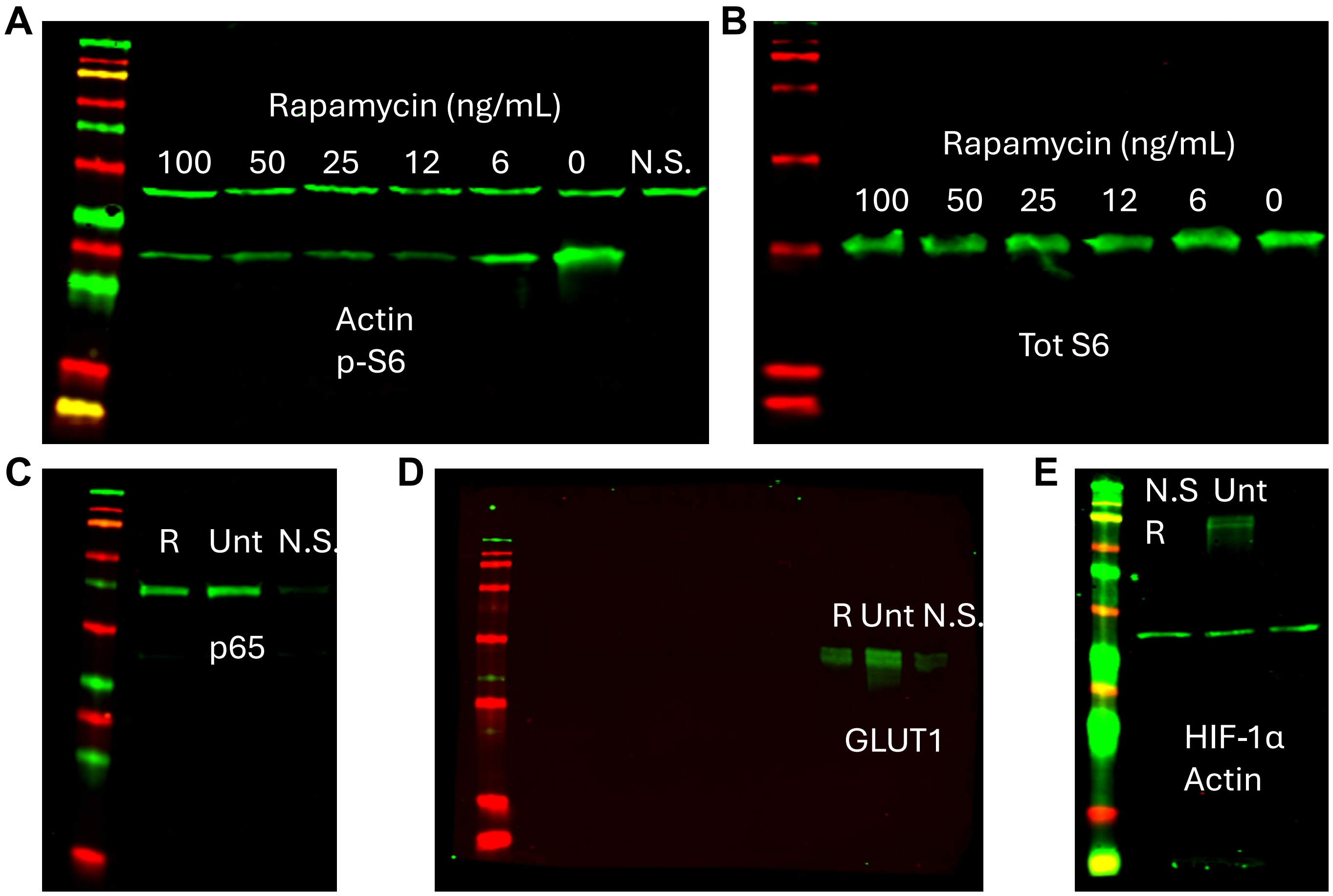

Figure S5. Western blots from *in vitro* signaling experiments. RAW264.7 murine macrophages were analyzed by Western blot for protein levels after activation with LPS and IFN-γ. **(A)** Pan actin (~45 kD band), p-S6 (~32 kD band); **(B)** total S6 protein (~32 kD band); **(C)** nuclear p65 (~65 kD band); **(D)** GLUT1 (~50 kD bands); **(E)** nuclear HIF-1a (~115 kD bands), pan actin (~45 kD band). R = rapamycin, Unt = untreated, N.S. = not stimulated with LPS and IFN-γ. Band size determined with Chameleon Duo Pre-stained Protein Ladder (LI-COR). Representative of at least 3 individual experiments.

Target	Conjugate	Vendor	ID	Use
CD11b	-	abcam	ab133357	IHC
GLUT1	-	Biorbyt	orb10725	IHC
Nitrotyrosine	-	EMD Millipore	AB5411	IHC
iNOS	-	abcam	ab15323	IHC
Rabbit IgG	biotin	Jackson IR	711-065-152	IHC
CD11b	BUV805	Thermo Fisher	M1/70	FC - Mouse
Ly-6G	APC/Fire 750	BioLegend	1A8	FC - Mouse
CD19	BV570	BioLegend	6D5	FC - Mouse
TCR beta	BUV395	Thermo Fisher	H57-597	FC - Mouse
NK1.1	PE/Cy7	BioLegend	PK136	FC - Mouse
CD45	PerCP/Cy5.5	BioLegend	30-F11	FC - Mouse
CD24	PE/Cy5	BioLegend	M1/69	FC - Mouse
CD64	APC	BioLegend	X54-5/7.1	FC - Mouse
MHCII	AF700	BioLegend	M5/114.15.2	FC - Mouse
CD16	PE/Cy7	BioLegend	3G8	FC - Human
CD15	BV711	BioLegend	W6D3	FC - Human
MPO	biotin	abcam	2D4	FC - Human
H3Cit	-	abcam	ab5103	FC - Human
Rabbit IgG	AF488	BioLegend	Poly4064	FC - Human
Pan actin	-	Cell Signaling	8456	WB
GLUT1	-	abcam	ab115730	WB
HIF-1α	-	Cell Signaling	14179	WB
p65	-	Cell Signaling	8242	WB
S6 (p-Ser235/236)	-	Cell Signaling	2211	WB
S6 (Total)	-	Cell Signaling	2217	WB
Rabbit IgG	800CW	LI-COR	925-32213	WB

Table S1. Antibodies used in immunohistochemistry, flow cytometry, and Western blot analyses.
